# Supplementary material for: Malayan kraits (Bungarus candidus) show affinity to anthropogenic structures in a human dominated landscape
Source: Sci Rep. 2022 May 3;12:7139. doi: 10.1038/s41598-022-11255-z (PMC9065047; doi:10.1038/s41598-022-11255-z)
Supplement: Supplementary file 1 — Supplementary Information. [file 41598_2022_11255_MOESM1_ESM.pdf]

Malayan kraits (*Bungarus candidus*) show affinity to anthropogenic structures in a human dominated landscape

Authors

Cameron Wesley Hodges[1*], Benjamin Michael Marshall[1], Jacques George Hill III[2], Colin Thomas Strine[1**]

Affiliations

[1]School of Biology, Institute of Science, Suranaree University of Technology, Muang Nakhon Ratchasima, Nakhon Ratchasima 30000, Thailand

[2]2444 Burns Hall, Science and Math Division, Northwest Arkansas Community College, Bentonville, Arkansas 72703, USA

Corresponding Author Email: [*]cameron.wesley.hodges@gmail.com, [**]strine.conservation@gmail.com

Supplemental Figures and Tables:

Supp. Table 1. Morphometric data, capture method, days tracked, and fate for each telemetered *B. candidus* individual

ID	Capture Method	SVL (mm)	TL (mm)	Mass (g)	Fate	Days Tracked
M01	Active Survey	1130	152	339.5	Mortality (road)	97.77
M02	Notification	1081	151	287.7	Transmitter failure	218.27
M07	Opportunistic	1013	141	248.4	Transmitter failure	138.98
M12	Notification	1303	153	544.3	Unable to recapture	156.35
M14	Notification	914	128	218	Unknown	74.96
F16	Notification	912	127	216.7	Unable to recapture	107.92
M22	Notification	650	94	63.6	Transmitter failure	48.88
M27	Active Survey	727	97	91.5	Transmitter failure	35.1
M28	Notification	772	114	91.2	Unable to recapture	100.92
M29	Notification	645	86	56.8	Transmitter failure	19.08
M32	Notification	1196	151	485	Unknown	100.66
M33	Notification	904	126	176.7	Unable to recapture	179.89
M35	Opportunistic	1113	139	450	Released	139.18
M36	Notification	1456	103	500	Unknown	59.89

Supp. Table 2. Table showing capture methods, capture dates, capture locations, release dates, release locations, and translocation distances upon release for every capture and implantation for each telemetered individual.

ID	Capture date	Capture Easting	Capture Northing	UTM Zone	Capture Method	Release date	Release Easting	Release Northing	UTM Zone	Distance (m)
M01	05-30-2018 22:59	179060	1646306	48	Active Survey	06-08-2018 19:44	179060	1646306	48	0
M01	09-11-2018 19:30	178813	1646489	48	Tracked to capture	09-12-2018 21:00	178813	1646489	48	0
M02	09-17-2018 00:00	179007	1647371	48	Notification	09-20-2018 23:00	179093	1647380	48	86.47
M07	10-19-2018 21:30	182020	1648494	48	Opportunistic capture	10-24-2018 23:30	182020	1648494	48	0
M12	11-16-2018 19:30	181180	1644862	48	Notification	11-21-2018 19:20	181286	1644926	48	123.82
M14	12-09-2018 20:13	180581	1646722	48	Notification	12-14-2018 20:30	180636	1646611	48	123.88
M02	12-25-2018 20:30	179062	1646598	48	Tracked to capture	01-02-2019 19:30	179092	1646681	48	88.26
F16	01-08-2019 07:20	179113	1646872	48	Notification	01-14-2019 23:00	179166	1646874	48	53.04
M07	02-08-2019 12:30	181904	1648459	48	Tracked to capture	02-21-2019 23:30	181892	1648457	48	12.17
M02	04-03-2019 20:08	179228	1646700	48	Tracked to capture	04-07-2019 22:30	179228	1646700	48	0
M22	04-23-2019 17:45	178776	1648029	48	Notification	04-30-2019 22:00	178783	1648067	48	38.64
M27	06-17-2019 20:25	177709	1647409	48	Active Survey	06-20-2019 21:00	177709	1647409	48	0
M28	07-03-2019 22:10	179260	1648429	48	Notification	07-08-2019 23:40	179210	1648287	48	150.55
M27	07-09-2019 21:15	177496	1646956	48	Tracked to capture	07-15-2019 22:30	177476	1646938	48	26.91
M29	07-25-2019 23:50	180146	1646557	48	Notification	07-30-2019 00:15	180338	1646437	48	226.42
M32	09-17-2019 23:50	822770	1647515	47	Notification	09-24-2019 20:40	822817	1647592	47	90.21
M33	09-18-2019 00:12	178254	1649809	48	Notification	09-24-2019 21:20	178225	1649861	48	59.54
M33	10-08-2019 20:25	178202	1650043	48	Tracked to capture	10-11-2019 23:50	178121	1650060	48	82.76
M35	10-11-2019 19:30	822301	1648030	47	Opportunistic capture	10-15-2019 23:59	822344	1648028	47	43.05
M33	11-03-2019 21:39	178188	1649925	48	Tracked to capture	11-04-2019 19:40	178121	1650060	48	150.71
M36	11-27-2019 20:42	177371	1647443	48	Notification	12-05-2019 18:40	177372	1647458	48	15.03

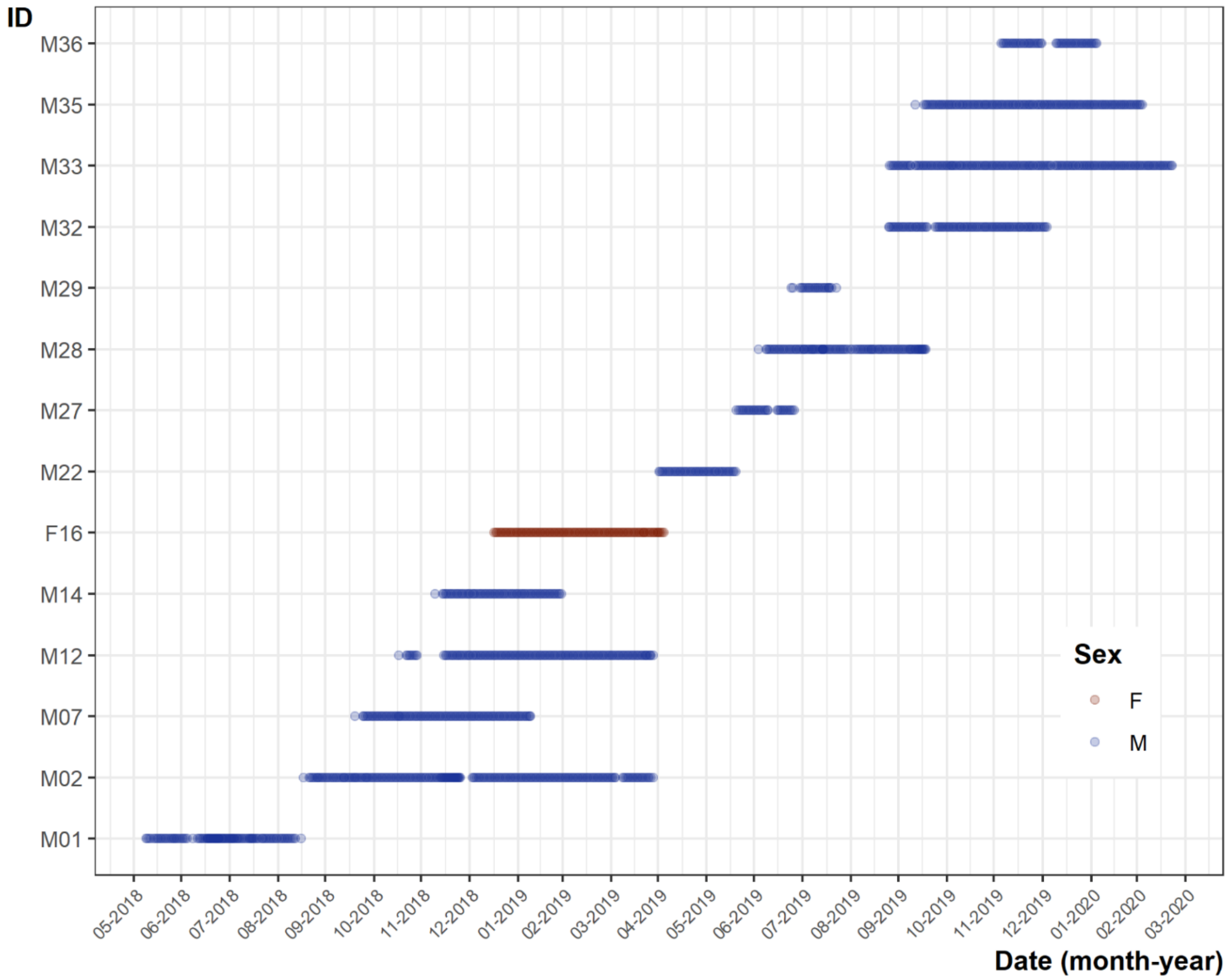

Supp. Figure 1. Completed location checks throughout the study period for males (semi-transparent blue) and the female (semi-transparent red), illustrating tracking durations and overlap of simultaneously tracked *B. candidus*.

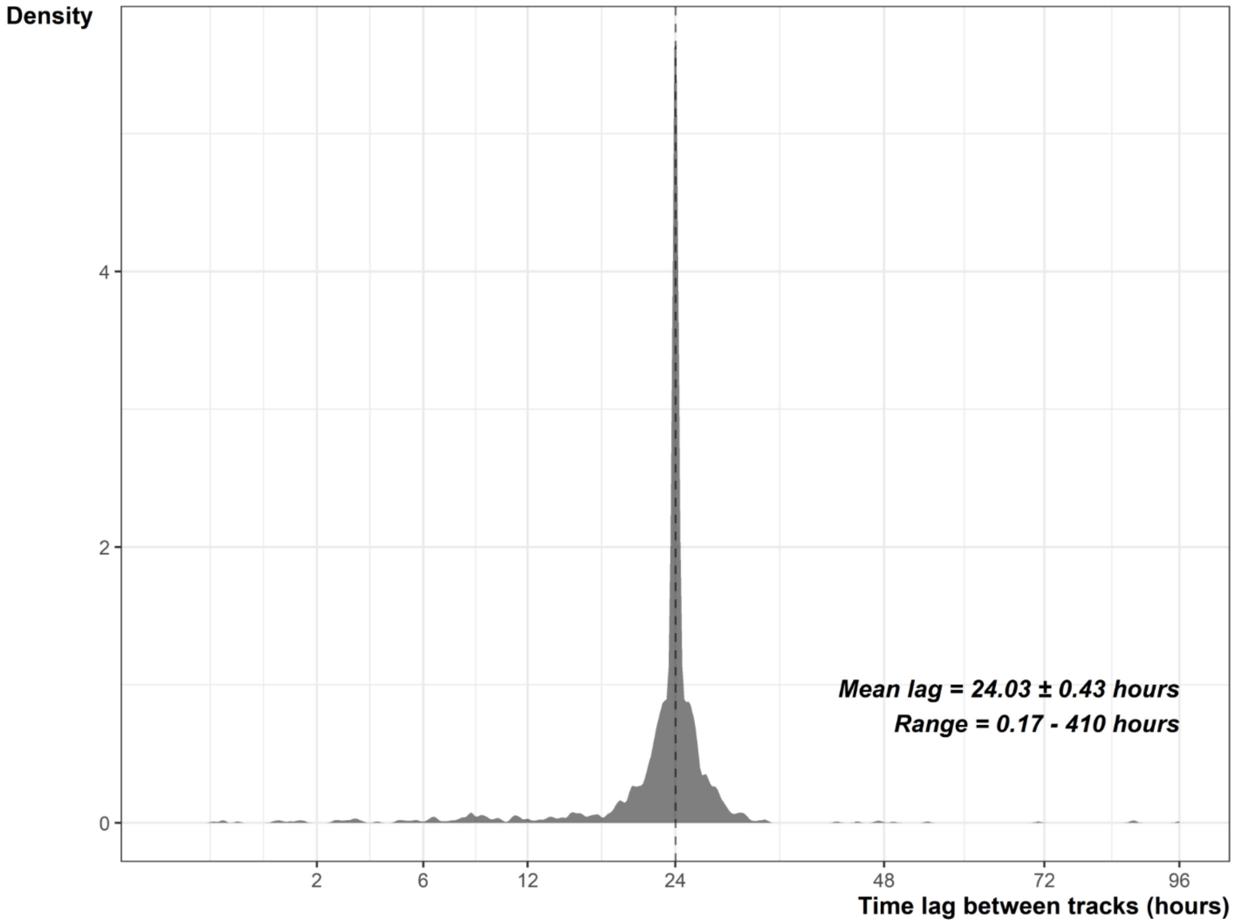

Supp. Figure 2. Density plot illustrating time-lags between tracks for all telemetered individuals.

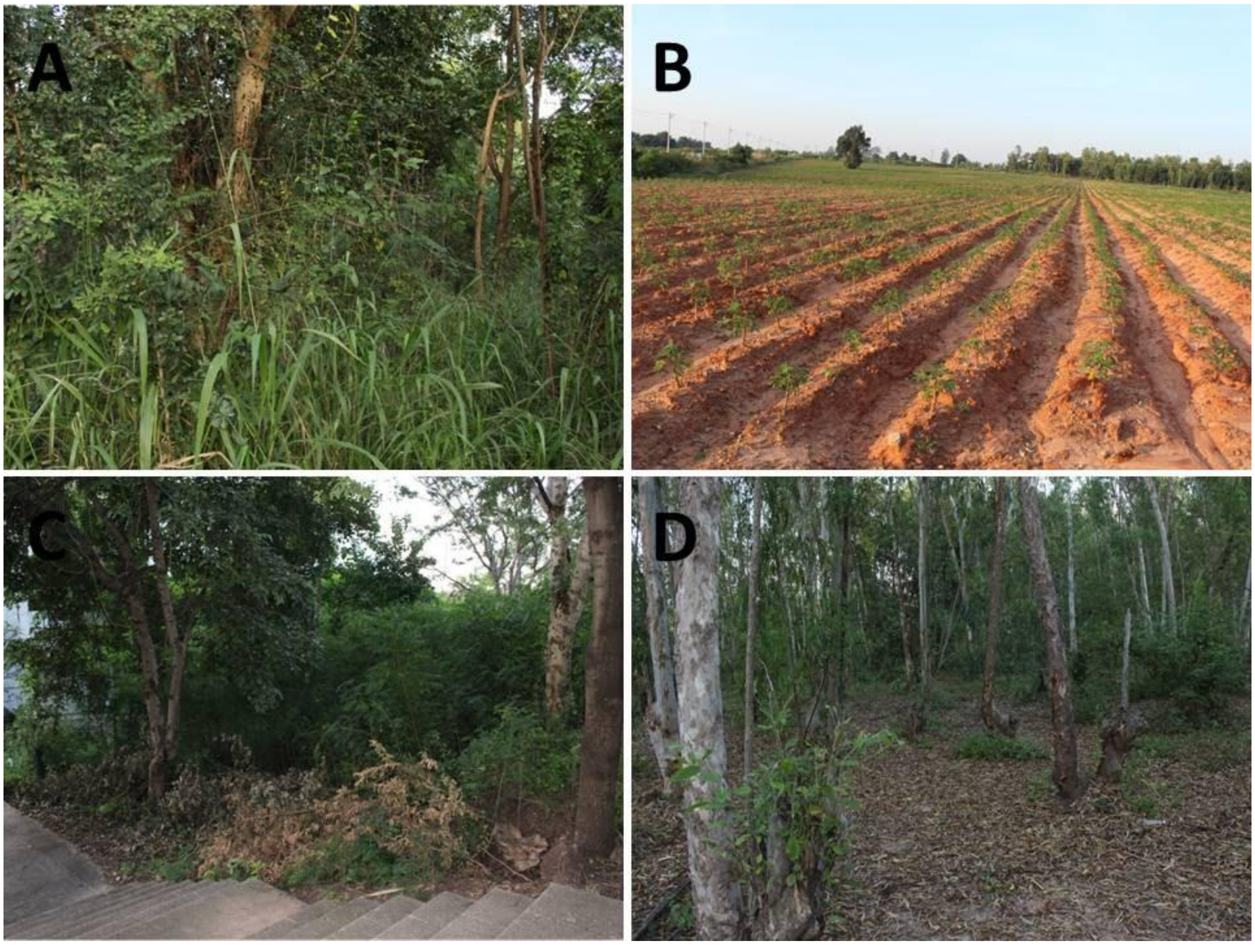

Supp. Figure 3. Photos of common land-use types within the study site a) Mixed deciduous forest b) Agriculture (cassava) c) Semi-natural area d) Plantation forest (Eucalyptus)

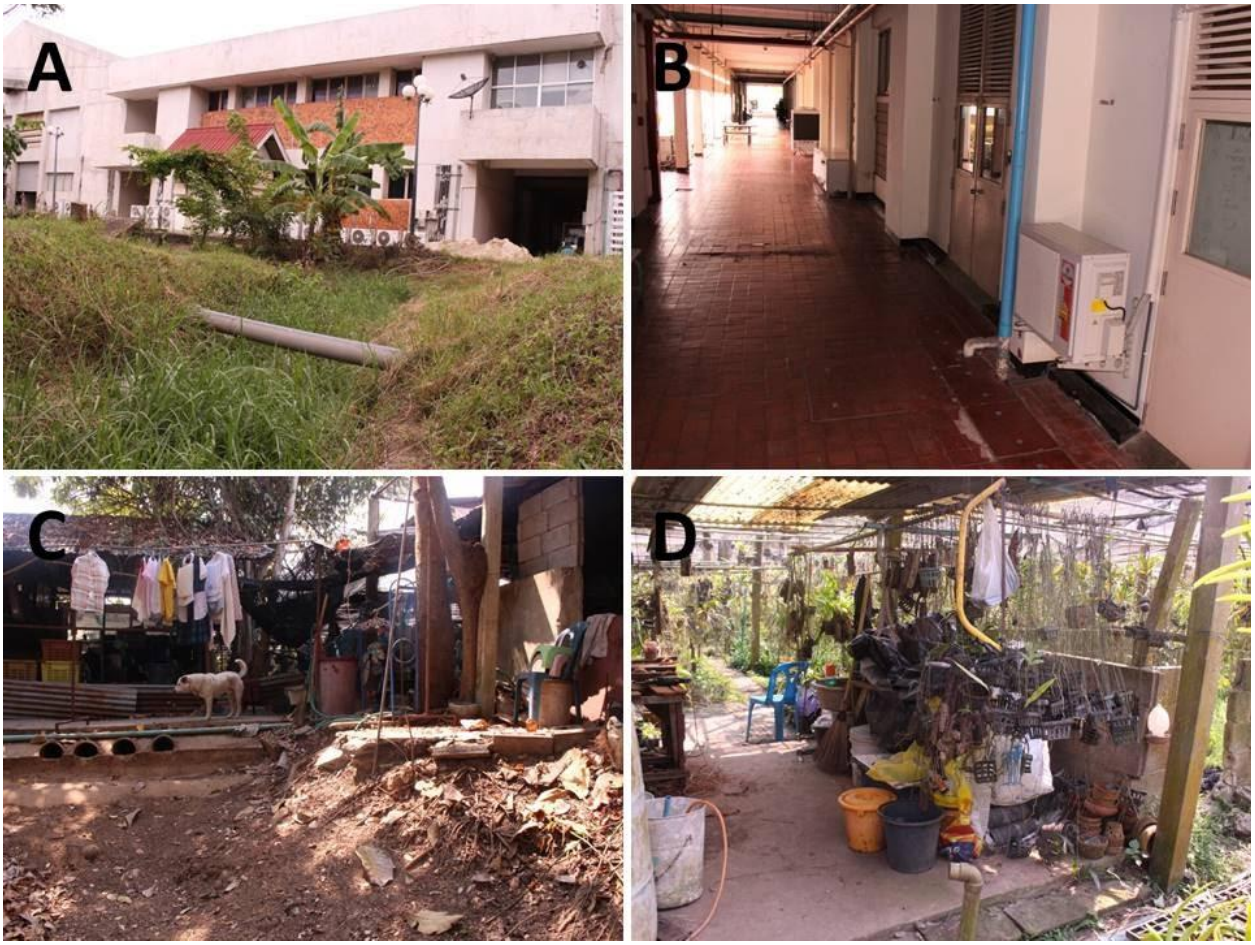

Supp. Figure 4. Examples *B. candidus* shelter sites among settlements a) Overgrown concrete drainage ditch outside a laboratory building b) Underneath the foundation or among the pipelines under a laboratory building c) Under a concrete at a residency d) Underneath a concrete floor of a gardening work-station

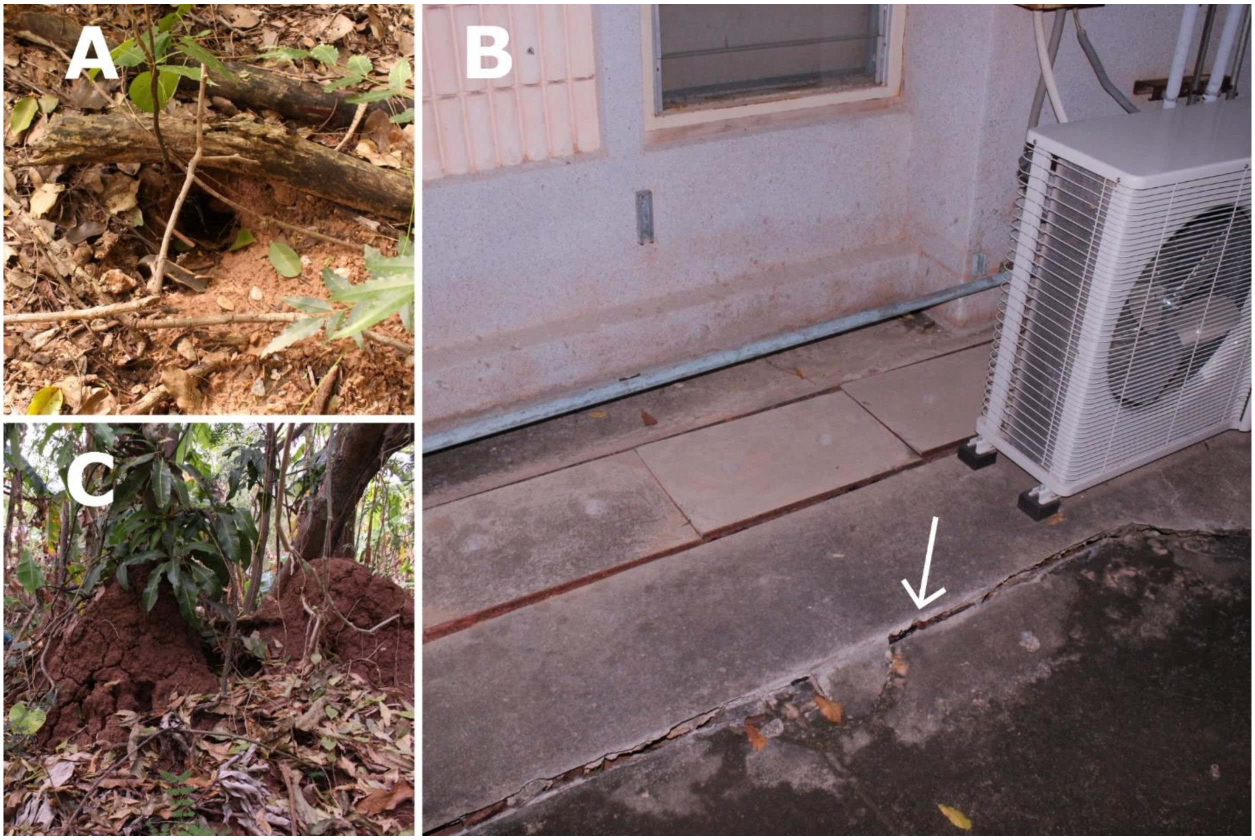

Supp. Figure 5. Examples of different shelter types most commonly used by *B. candidus* a) Burrow b) Anthropogenic - under concrete c) Termite mound

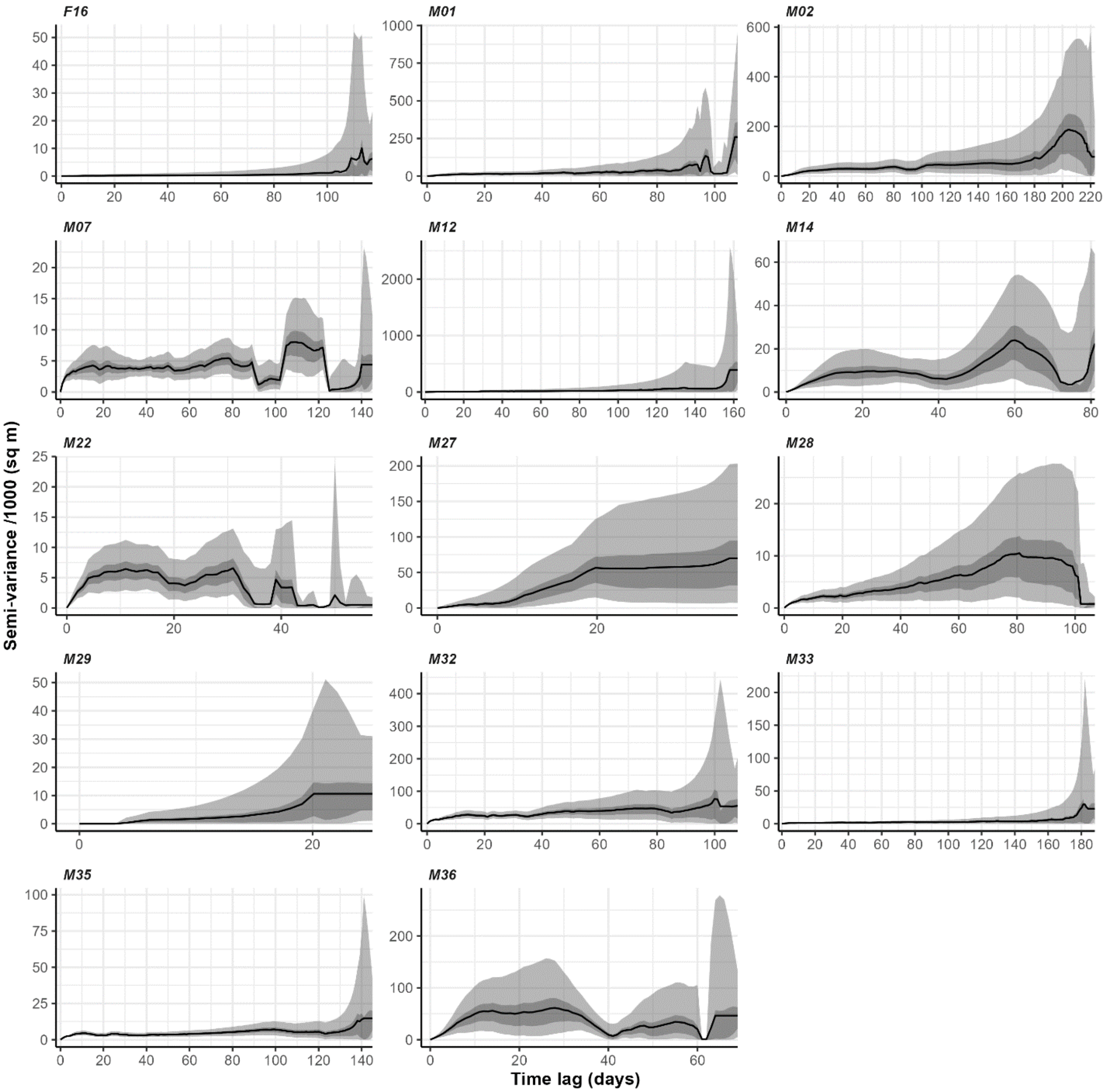

Supp. Figure 6. Variogram plots for AKDEs.

Supp. Table 3. Movement and space use summary data for each telemetered *B. candidus*. Dynamic Brownian Bridge Movement model 90%, 95%, and 99% confidence areas (hectares), mean motion variance (σ^2m), mean movement distance (MMD, in meters), mean daily displacement (MDD, in meters), and relocation probabilities (i.e., proportion of daily fixes moved) shown with overall averages for males (n = 13) (note M29 excluded from dBBMM occurrence distribution and motion variance means, n = 12).

ID	90% (ha)	95% (ha)	99% (ha)	σ^2m	MMD (m)	MDD (m)	Move probability
M01	6.73	10.57	18.8	1.23 ± 0.17	113.7 ± 26.51	38.35 ± 10.53	0.34
M02	7.7	12.91	25.68	1.2 ± 0.19	85.18 ± 12.2	23.17 ± 4.20	0.27
M07	2.16	5.58	23.39	2.37 ± 0.53	132.87 ± 25.14	24.11 ± 6.38	0.18
M12	3.11	5.04	8.4	0.66 ± 0.08	165.39 ± 46.02	27.01 ± 9.0	0.16
M14	1.06	1.52	2.52	0.55 ± 0.06	93.53 ± 16.76	21.03 ± 5.8	0.22
F16	0.03	0.05	0.42	0.16 ± 0.04	47.15 ± 11.75	5.68 ± 2.0	0.12
M22	3.96	6.05	10.07	2.33 ± 0.34	69.85 ± 16.03	37.0 ± 9.6	0.52
M27	2.79	3.63	5.33	0.6 ± 0.2	80.86 ± 33.93	19.62 ± 10.11	0.24
M28	1.89	2.77	4.81	0.58 ± 0.09	66.45 ± 10.68	19.22 ± 4.26	0.29
M29	0.03	0.04	0.06	na	91.67 ± 79.67	25.0 ± 22.65	0.27
M32	29.8	56.2	119.55	9.63 ± 2.34	259.65 ± 57.67	60.99 ± 17.37	0.23
M33	1.61	3.11	6.46	0.7 ± 0.11	82.77 ± 10.44	14.54 ± 2.98	0.18
M35	3.39	6.79	13.09	1.25 ± 0.21	160.17 ± 19.66	20.59 ± 5.17	0.13
M36	15.71	20.47	29.85	4.38 ± 0.52	185.44 ± 46.23	55.98 ± 18.04	0.3
Avg (males)	*6.66 ± 2.41*	*11.22 ± 4.37*	*22.33 ± 9.21*	*1.70 ± 0.18*	*117.78 ± 8.23*	*27.48 ± 2.36*	*0.23*

Supp. Table 4. Results from AKDE analysis, including effective sample size (DOF_area), the type of top fitted model (movMod), the 95% contour AKDE area estimates (ha) with 95% CI, the speed estimate with 95% CI is calculable for the top movement model (m/day).

ID	DOF area	Move model	aKDE low	aKDE est	aKDE high	Speed low	Speed est	Speed high
F16	3.87	OU anisotropic	0.62	2.33	5.17	0	Inf	Inf
M01	3.06	OUF anisotropic	17.29	82.07	196.32	94.3	106.36	118.77
M02	5.49	OUF anisotropic	22.85	65.94	131.45	93.58	138.94	188.47
M07	55.22	OU isotropic	4.03	5.34	6.84	0	Inf	Inf
M12	1.49	OUF anisotropic	0.35	4.99	15.58	62.19	68.36	74.68
M14	2.19	OU anisotropic	2.52	18.19	48.99	0	Inf	Inf
M22	19.98	OU anisotropic	2.7	4.42	6.56	0	Inf	Inf
M27	1.87	OU anisotropic	1.51	13.93	39.83	0	Inf	Inf
M28	135.75	IID anisotropic	2.75	3.28	3.86	0	Inf	Inf
M29	1.9	OUf anisotropic	0.54	4.83	13.73	12.85	16.76	20.92
M32	14.12	OU anisotropic	35.7	65.1	103.19	0	Inf	Inf
M33	15.56	OU isotropic	3.9	6.89	10.7	0	Inf	Inf
M35	39.38	OUF anisotropic	5.46	7.67	10.24	71.68	82.81	94.32
M36	5.65	OUF anisotropic	31.16	88.23	174.49	122.63	153.94	186.92

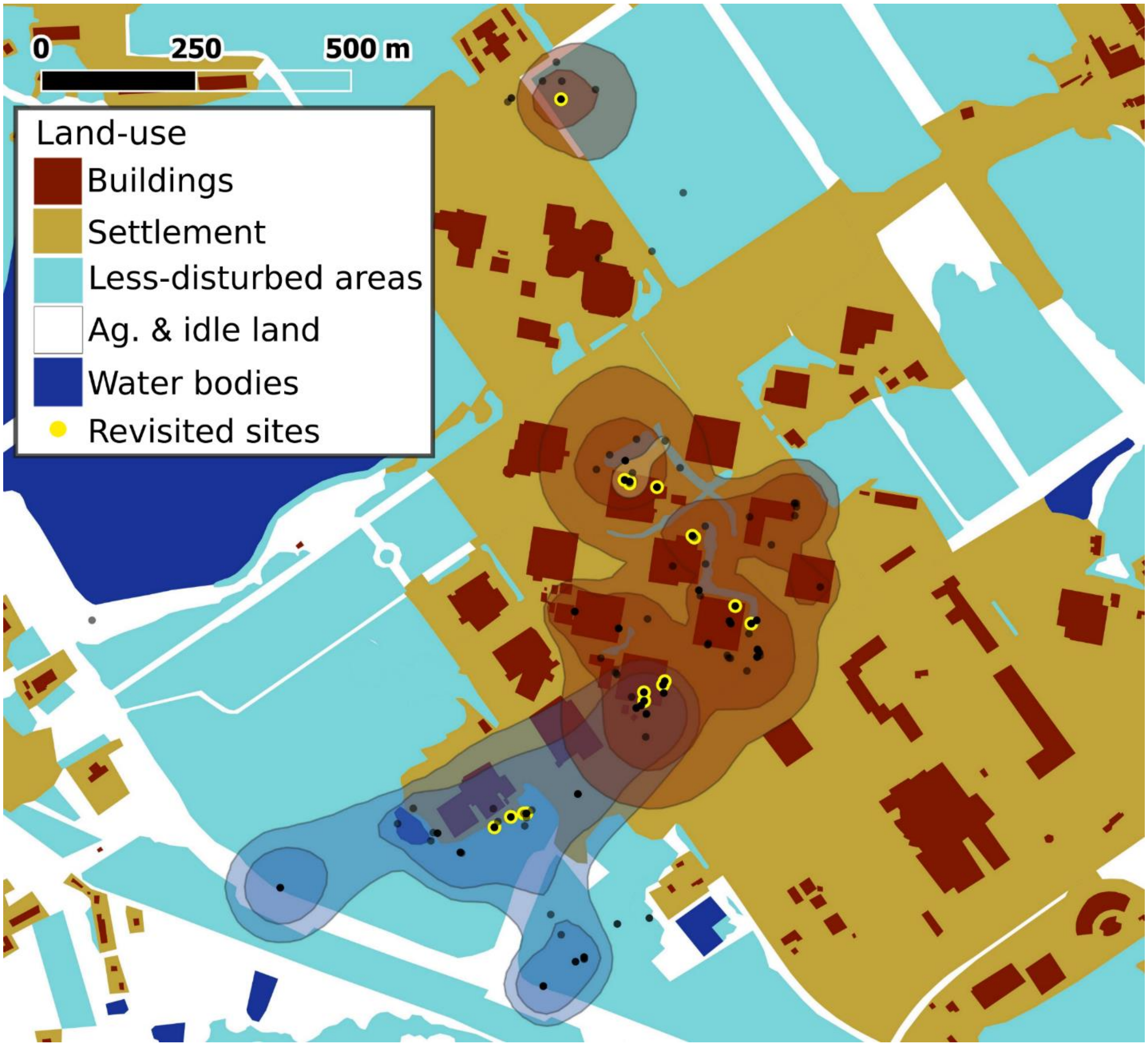

Supp. Figure 7. 95% and 99% dBBMM occurrence distribution confidence areas for M01 (blue), M02 (red), and F16 (tan) with revisited sites marked in yellow.

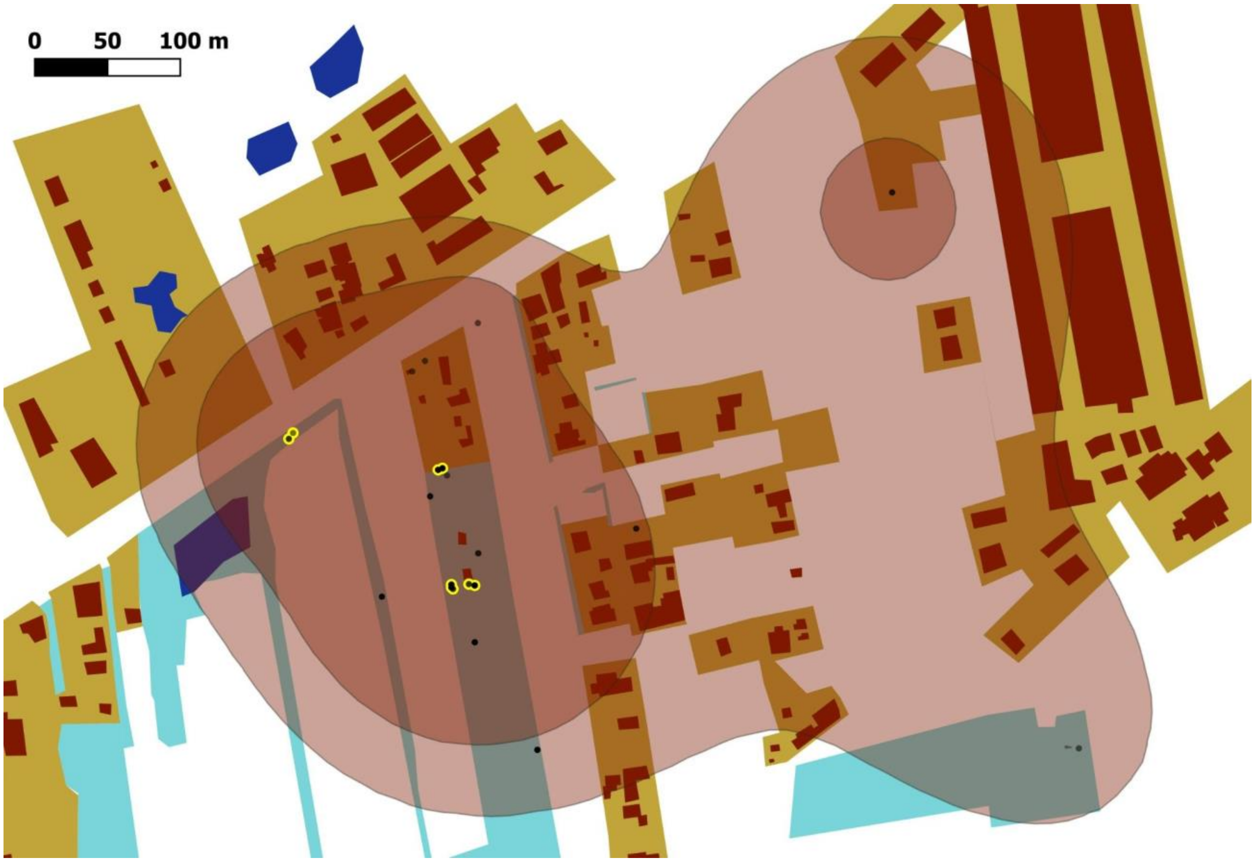

Supp. Figure 8. 95% and 99% dBBMM occurrence distribution confidence areas for M07 (red) with revisited sites marked in yellow.

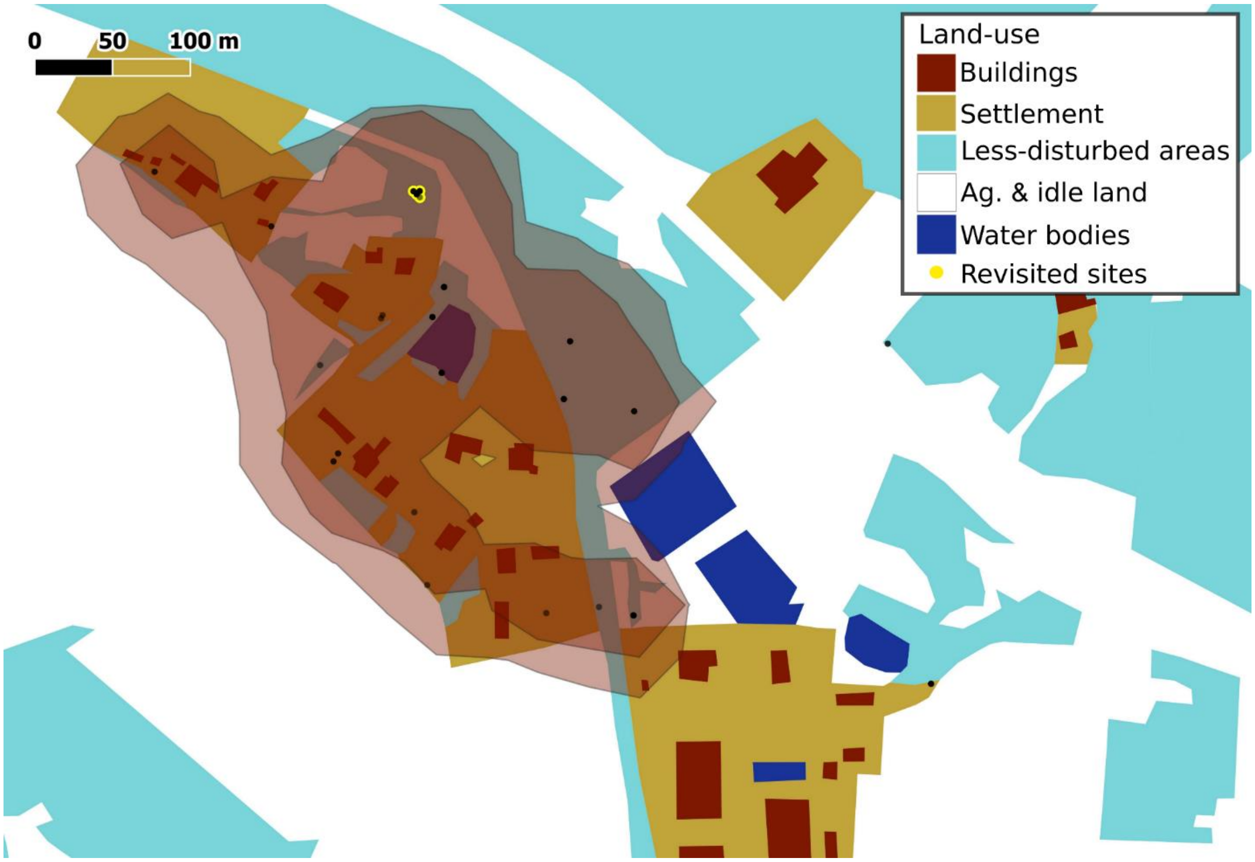

Supp. Figure 9. 95% and 99% dBBMM occurrence distribution confidence areas for M12 (red) with revisited sites marked in yellow.

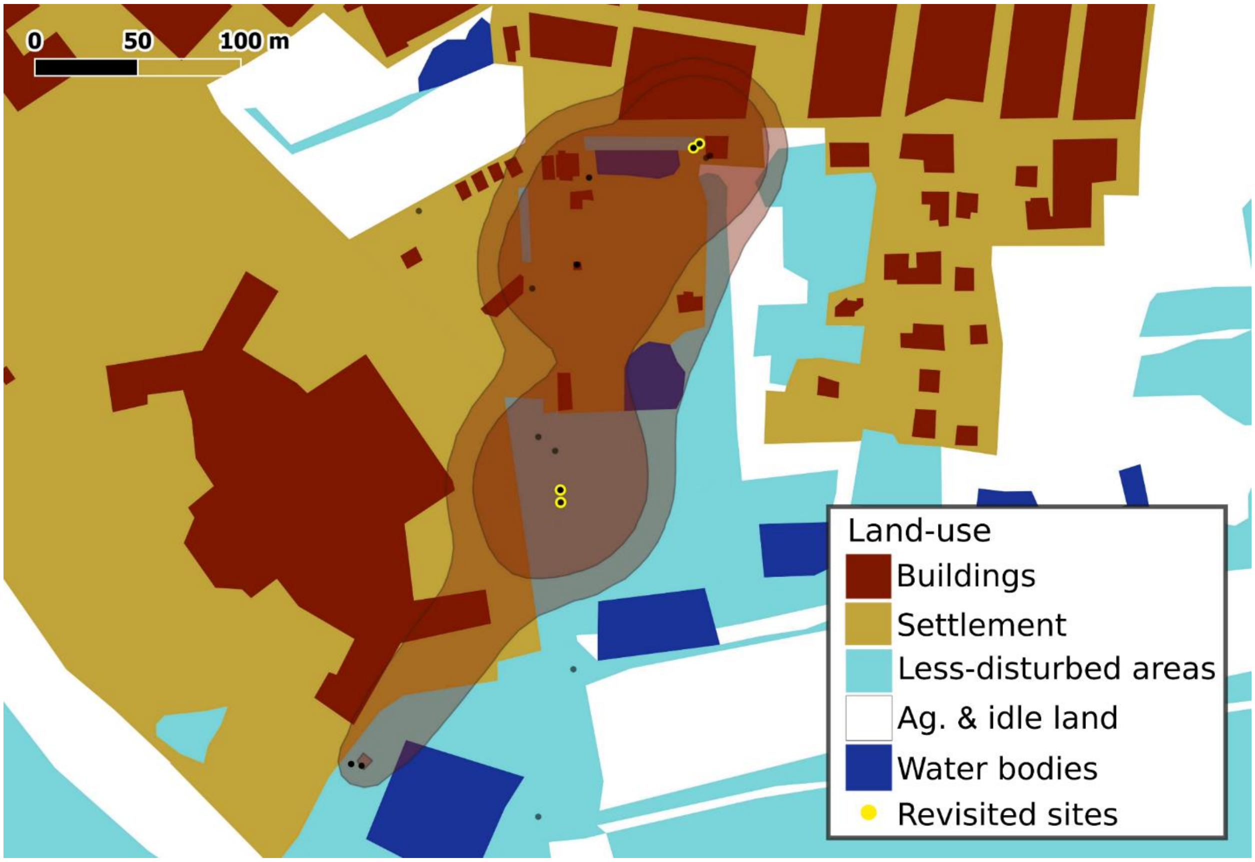

Supp. Figure 10. 95% and 99% dBBMM occurrence distribution confidence areas for M14 (red) with revisited sites marked in yellow.

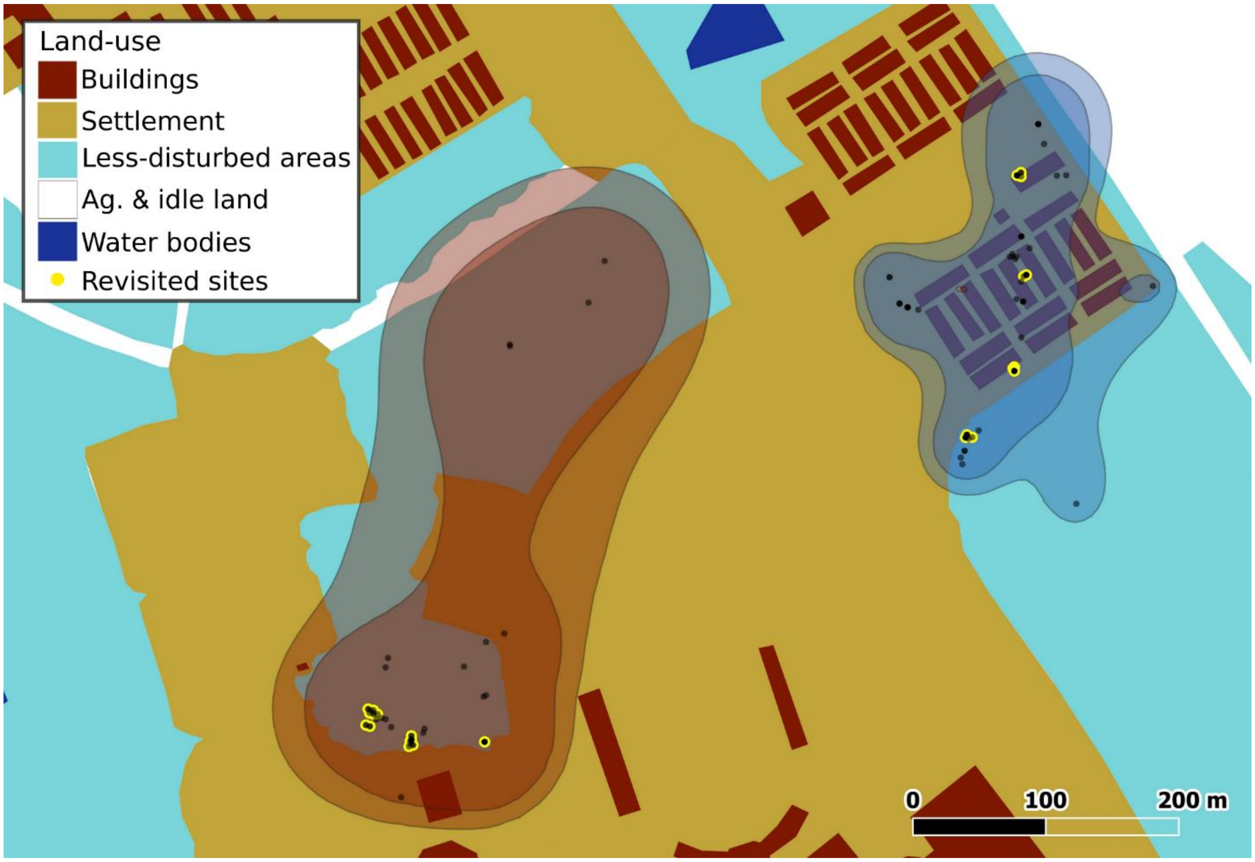

Supp. Figure 11. 95% and 99% dBBMM occurrence distribution confidence areas for M22 (red) and M28 (blue) with revisited sites marked in yellow.

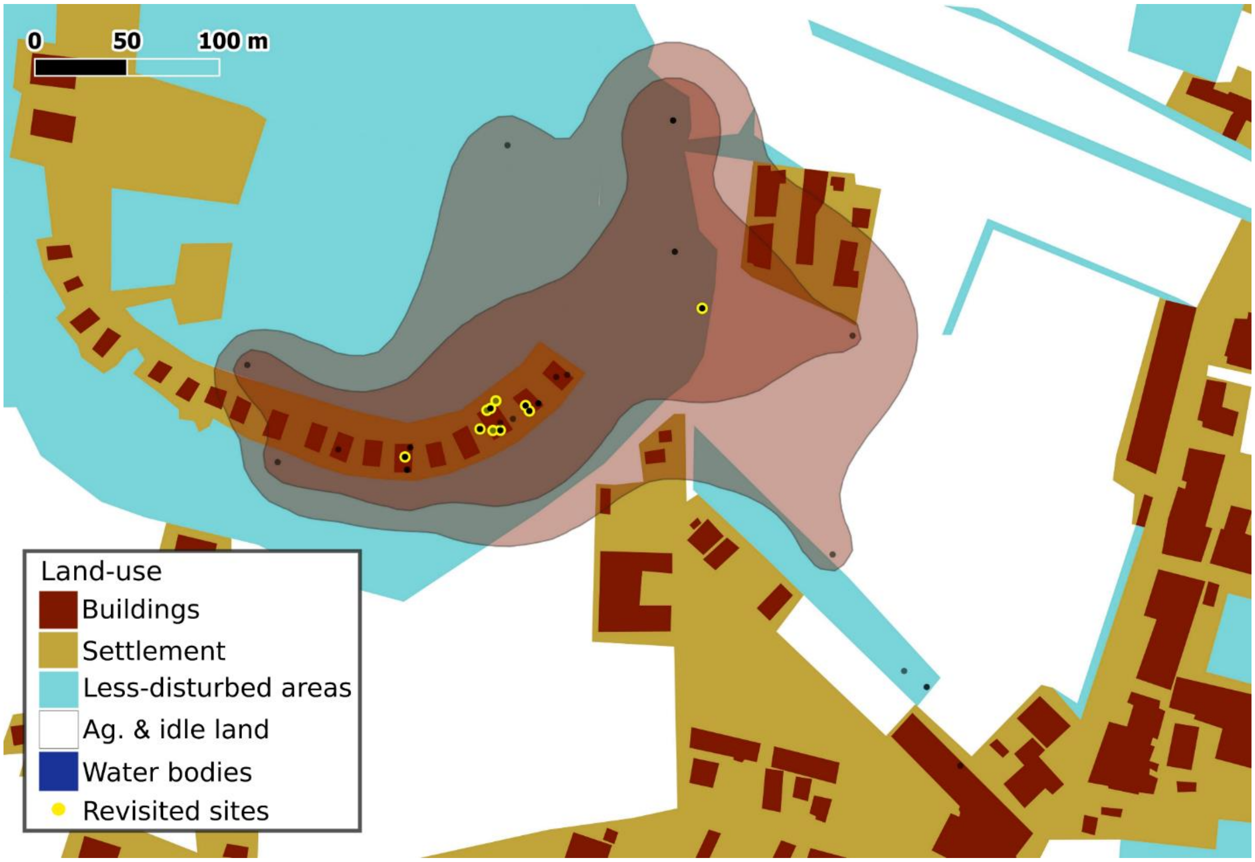

Supp. Figure 12. 95% and 99% dBBMM occurrence distribution confidence areas for M33 (red) with revisited sites marked in yellow.

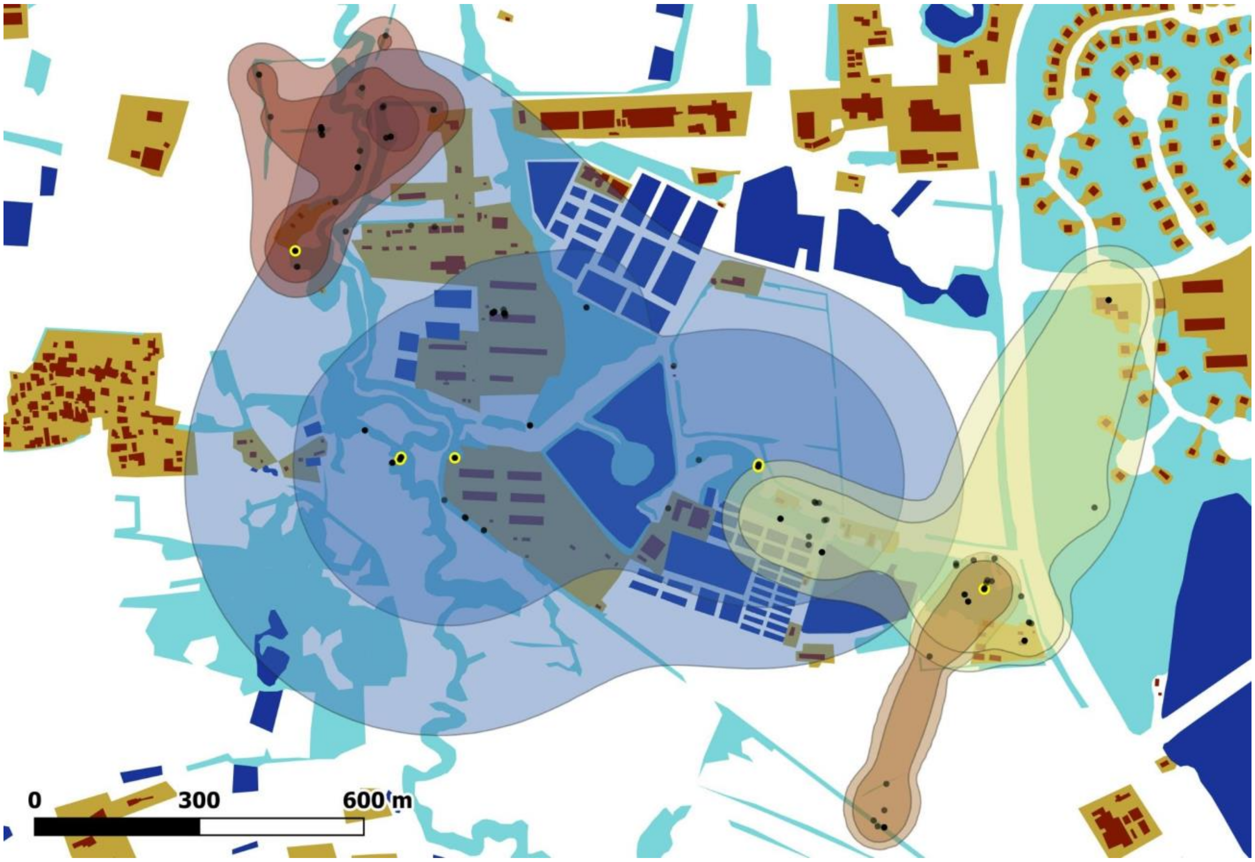

Supp. Figure 13. 95% and 99% dBBMM occurrence distribution confidence areas for M27 (orange), M32 (blue), M35 (red), and M36 (yellow) with sites revisited marked in yellow.

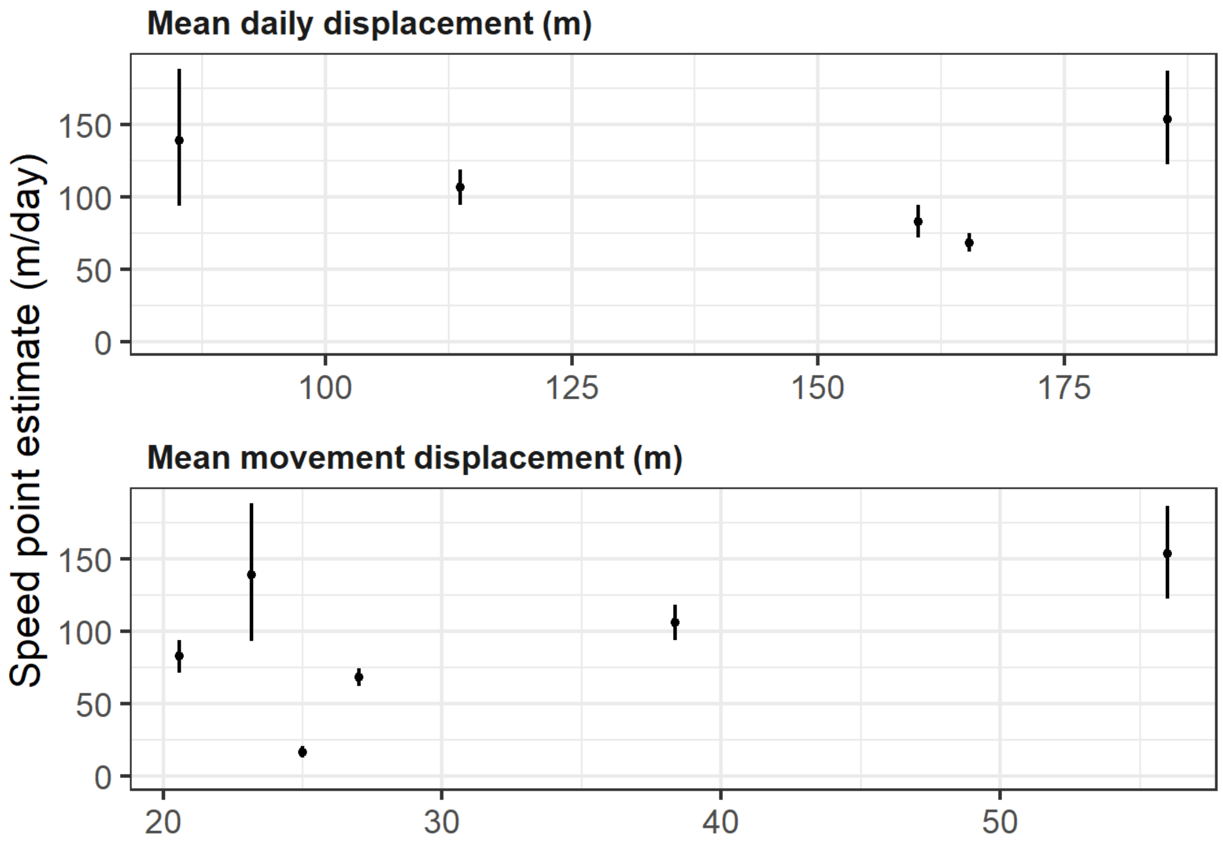

Supp. Figure 14. The relationship between mean daily displacement and mean movement displacement and the speed estimates extracted from movement models. Error bars show the 95% CI surrounding the speed estimates. Not all individuals could have speed estimated because of the failure to successfully fit movement models.

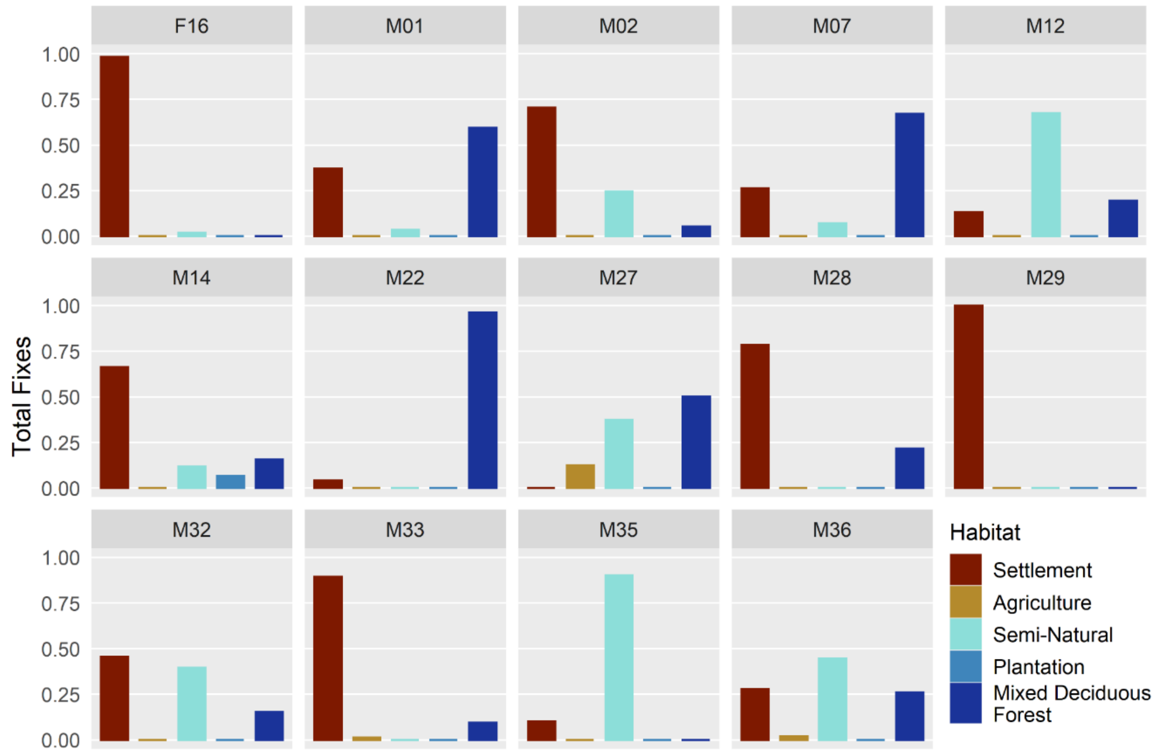

Supp. Figure 15. Habitat use proportions for each telemetered individual

Supp. Table 5. Total number of fixes an individual was located in each land-use type. MDF = mixed deciduous forest.

Snake ID	Settlement	Semi-Nat	Agriculture	MDF	Plantation
M01	43	4	0	69	0
M02	162	56	0	12	0
M07	34	9	0	87	0
M12	19	97	0	28	0
M14	51	9	0	12	5
F16	110	2	0	0	0
M22	2	0	0	49	0
M27	0	12	4	16	0
M28	91	0	0	25	0
M29	24	0	0	0	0
M32	45	39	0	15	0
M33	160	0	2	17	0
M35	14	127	0	0	0
M36	15	24	1	14	0
Total	*770 (51.2%)*	*379 (25.2%)*	*7 (0.5%)*	*344 (22.8%)*	*5 (0.3%)*

Supp. Table 6. Total number of fixes an individual was located within each shelter type

ID	Burrow	Termite Md	Anthro	Unk
M01	27	20	40	5
M02	72	12	135	2
M07	116	7	2	3
M12	67	62	8	6
M14	41	1	9	25
F16	0	1	108	1
M22	44	6	0	0
M27	27	0	0	2
M28	70	4	33	0
M29	0	0	20	1
M32	42	1	44	10
M33	23	5	148	0
M35	36	95	0	9
M36	17	3	12	21
Total	*582 (40.3%)*	*217 (15%)*	*559 (38.7%)*	*85 (5.9%)*

Supp. Table 7. Model formulas and AIC scores for individual ISSF models. * with emboldened text indicates AIC scores within < 2 Δ AIC of the model that best supports the observed movements for an individual

model formula	M01	M02	M07	M12	M14	M22	M27	M28	M32	M33	M35	M36
model1 (null)	345.11	620.56	249.4	239.03	175.24	300.2	75.62	358	259.89	310.62	185.6	163.86
Settlement	346.5	626.17	254.49	225.74	180.15	298.2	75.7	357.73	259.84	290	190.98	160.5
Road	350.5	615.23	251.22	239.16	180.62	305.21	77.42	360.64	260.81	300.81	190.45	168.36
Building	346.56	609.77	248.64	231.38	180.83	305.39	77.44	**352.13***	255.76	**281.73***	191.17	**157.75***
Agriculture	349.89	625.41	237.47	244.6	180.25	303.19	80.15	362.44	264.66	313.7	189.9	168.79
Natural	341.78	618.91	236.46	226.38	172.86	277.19	77.44	363.57	**250.69***	311.44	**157.83***	161.25
Ag + Nat + Build	**337.58***	607.53	**231.35***	**205.51***	**168.46***	**273.55***	**74.51***	**351.51***	255.29	291.22	**157.72***	**157.3***
Rd + Build + Nat	**338.52***	**597.76***	238.29	**206.6***	**167.11***	**273.97***	**73.41***	**351.47***	254.87	**282.67***	**157.91***	**156.44***
Rd + Ag + Nat	343.87	612.42	**232.19***	224.75	170.85	275.56	76.53	360.74	263.23	288.14	**157.69***	162.56

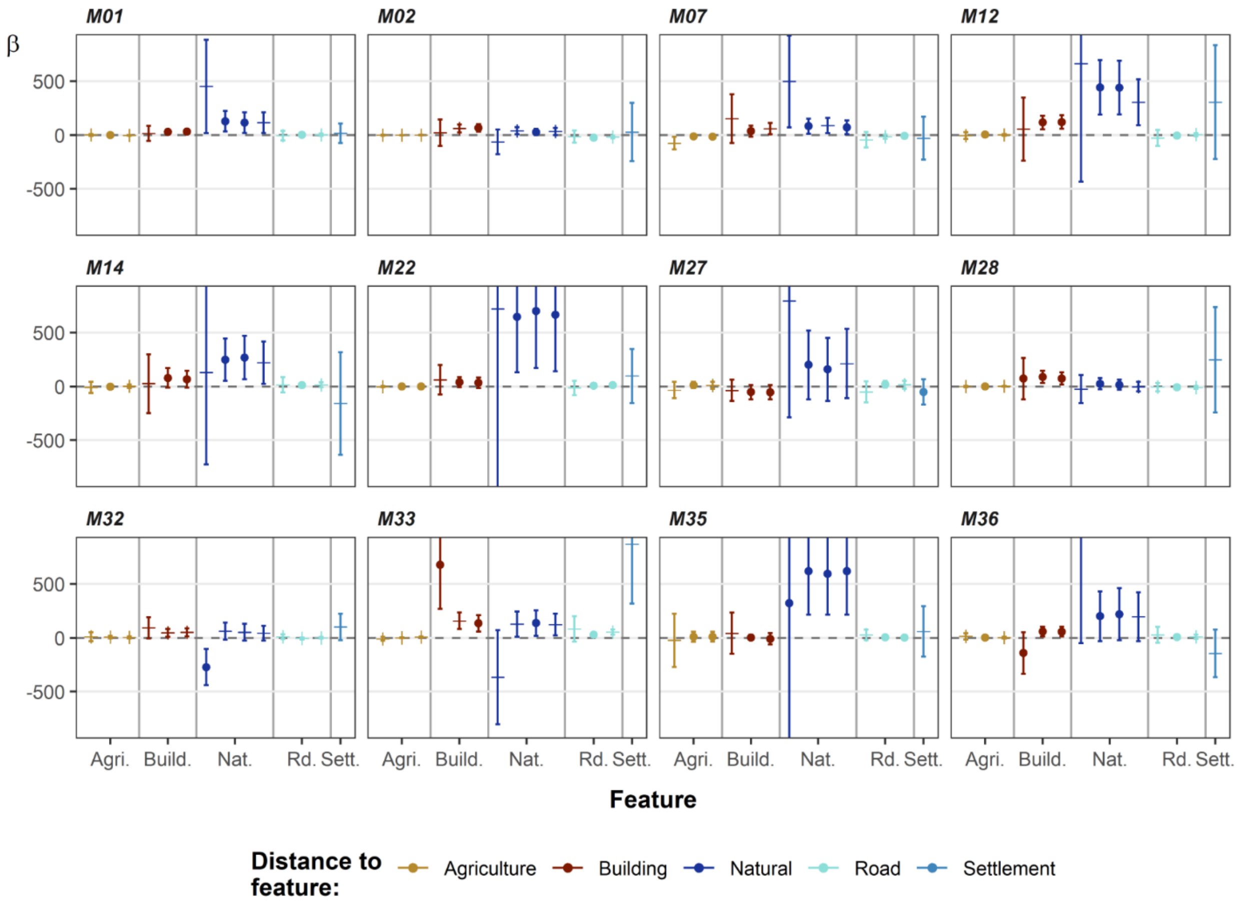

Supp. Figure 16. Individual ISSF model results based on distance to habitat features. Positive estimates suggest association with habitat feature, error bars indicate 95% confidence intervals, and circles mark the habitat features that were included in models with AIC scores within <2 D AIC of top performing models.

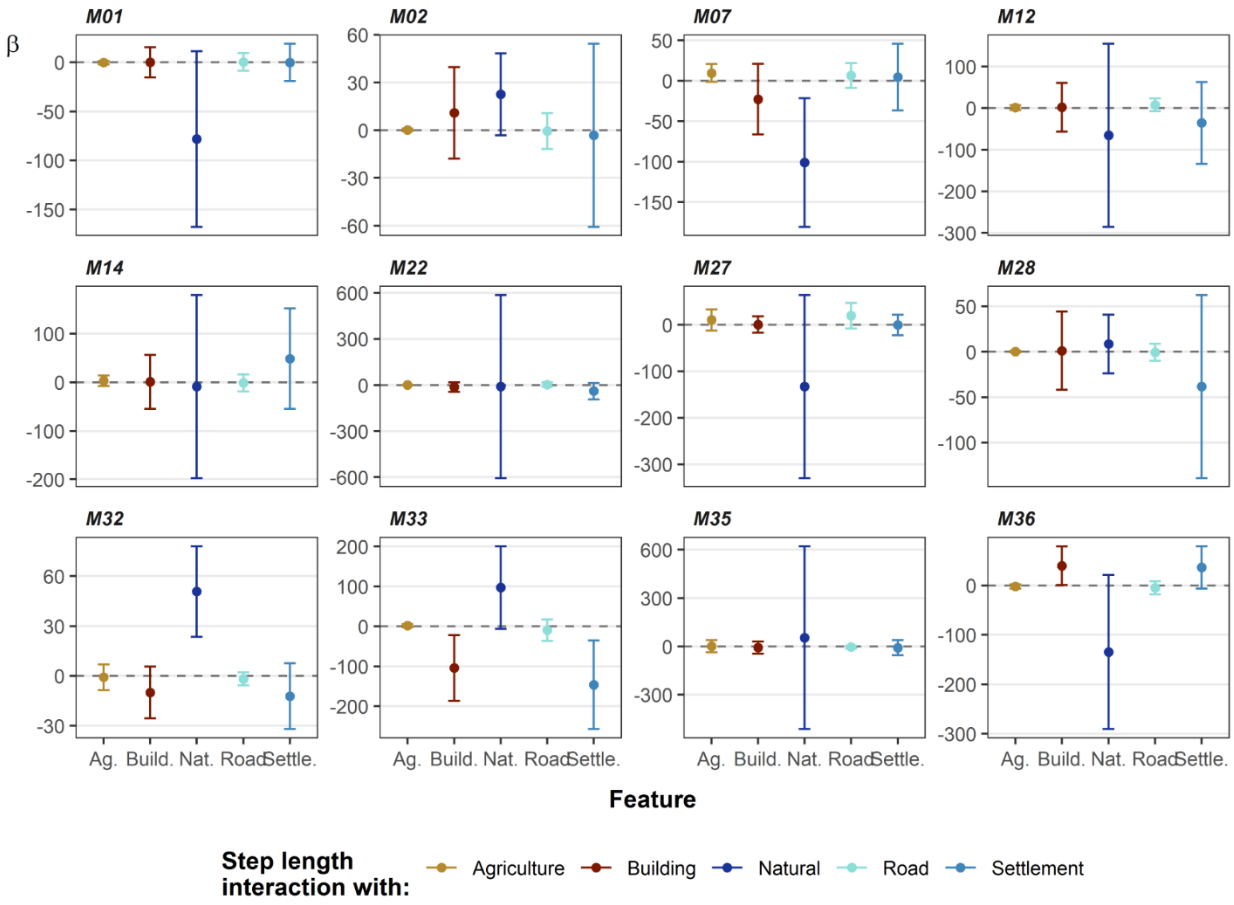

Supp. Figure 17. Individual ISSF model results based on the interaction between land-use features and step length. Error bars indicate 95% confidence intervals

Supp. Table 8. Mean daily displacement (MDD), movement distance (MMD), and movement probabilities (mv prob) calculated for each individual during each season as well as overall

ID	Cold MDD (m)	Hot MDD (m)	Wet MDD (m)	MDD (m)	Cold mv prob	Hot mv prob	Wet mv prob	Mv prob
M01	na	na	38.35 ± 10.53	38.35 ± 10.53	na	na	0.34	0.34
M02	24.13 ± 6.36	16.51 ± 4.45	67.2 ± 27.64	23.17 ± 4.20	0.27	0.2	0.8	0.27
M07	29.61 ± 8.02	4.64 ± 4.42	na	24.11 ± 6.38	0.22	0.04	na	0.18
M12	33.71 ± 21.45	22.73 ± 5.57	na	27.01 ± 9.0	0.11	0.19	na	0.16
M14	20.42 ± 7.5	22.07 ± 9.24	na	21.03 ± 5.8	0.23	0.21	na	0.22
F16	11.56 ± 9.01	4.67 ± 1.77	na	5.68 ± 2.0	0.13	0.12	na	0.12
M22	na	22.61 ± 5.87	60.47 ± 22.72	37.0 ± 9.6	na	0.48	0.58	0.52
M27	na	na	19.62 ± 10.11	19.62 ± 10.11	na	na	0.24	0.24
M28	0	na	23.39 ± 5.08	19.22 ± 4.26	0	na	0.35	0.29
M29	na	na	25.0 ± 22.65	25.0 ± 22.65	na	na	0.27	0.27
M32	42.44 ± 15.25	na	302.14 ± 111.85	60.99 ± 17.37	0.19	na	0.86	0.23
M33	15.81 ± 3.65	6.27 ± 3.12	61.17 ± 39.48	14.54 ± 2.98	0.19	0.1	0.5	0.18
M35	26.94 ± 6.66	0	na	20.59 ± 5.17	0.17	0	na	0.13
M36	60.55 ± 19.38	0	na	55.98 ± 18.04	0.33	0	na	0.3
Male Means	*28.71 ± 3.56*	*14.78 ± 2.1*	*41.34 ± 6.28*	*27.48 ± 2.36*	*0.21*	*0.18*	*0.38*	*0.23*
